# Experimental models for subclinical hypocalcemia and endometritis induction in cattle: a literature review

**DOI:** 10.1590/1984-3143-AR2024-0143

**Published:** 2025-12-04

**Authors:** Jerbeson Hoffmann da Silva, André Gustavo Cabrera Dalto, Eduardo Schmitt, Bernardo Garziera Gasperin, Carlos Bondan, Monique Tomazele Rovani

**Affiliations:** 1 Setor de Grandes Ruminantes, Universidade Federal do Rio Grande do Sul – UFRGS, Porto Alegre, RS, Brasil; 2 Programa de Pós-graduação em Veterinária, Universidade Federal de Pelotas – UFPEL, Pelotas, RS, Brasil; 3 Programa de Pós-graduação em Bioexperimentação, Universidade de Passo Fundo – UPF, Passo Fundo, RS, Brasil

**Keywords:** subclinical endometritis, subclinical hypocalcemia, induction of endometritis, induction of hypocalcemia

## Abstract

Serum calcium fluctuations are common during the peripartum period of dairy cattle and several studies have attempted to demonstrate the impact of decreased blood calcium (Ca) on subclinical endometritis; however, the highly dynamic and complex nature of the peripartum period in dairy cows may impair the establishment of the cause-and-effect relationship. The objective of this review is to compile information regarding hypocalcemia and subclinical endometritis and their relationship, as well as the available in vivo and in vitro study models that artificially induce subclinical states of hypocalcemia and endometritis in cows that are not in peripartum period. Regarding hypocalcemia, several studies have demonstrated the effectiveness and safety of protocols using Ca chelators such as ethylenediaminetetraacetic acid (EDTA) or ethylene glycol tetraacetic acid (EGTA) in vivo. The induced transitory hypocalcemia impaired feed intake, rumination and neutrophilic phagocytic and oxidative burst response. However, the effects on uterine environment remain poorly explored. Although these experimental models allow the understanding of the effects of hypocalcemia alone, without the peripartum metabolic and hormonal variations, the effects are likely underestimated because dairy cows may experience hypocalcemia for much longer periods. For studying bovine endometritis, the main experimental in vivo model is the intrauterine infusion of pathogenic bacteria or their components (lipopolysaccharide - LPS), which induce endometrial inflammation, even causing long-term negative effects. Several in vitro and ex vivo models have also been developed, which are mainly indicated to investigate the mechanisms underlying endometrial inflammation in cattle because there is no interaction with other tissues, organs and systems, as would occur in vivo. In conclusion, current models still face limitations and, therefore, future efforts to the development and refinement of in vivo and in vitro experimental models are necessary.

## Introduction

The peripartum period imposes substantial challenges for calcium (Ca) homeostasis in dairy cattle, as requirement at calving increases to 30-50 g/day for colostrum production, an amount that can represent nine times the usual serum Ca levels (Horst et al., 2005). Calcemia in cattle needs to be maintained between 8.5 and 10 mg/dL (Goff, 2004) for proper vital functions, which can be a major challenge in this period. Ca is vital for processes such as neurotransmission, muscle contraction, metabolism, cell growth and proliferation, activation and migration of leukocytes; for proper endocrine and metabolic functioning; and for the normal composition of bones and teeth (Goff, 2000, 2004; Martinez et al., 2012; Martinez et al., 2014; Goff 2018).

It is estimated that the incidence of subclinical hypocalcemia (SCH) is approximately 25% in primiparous cows and 50% in multiparous cows (Reinhardt et al., 2011). As Ca acts on muscle contraction, inflammation and immune function, it is a key component in the occurrence of some metabolic and infectious disorders during the postpartum period (Kimura et al., 2006) and several studies have suggested a relationship between Ca levels and the occurrence of clinical or subclinical endometritis (Martinez et al., 2012, 2014; Ruiz-García et al., 2022).

Clinical endometritis is characterized by the presence of purulent vaginal discharge 21 days postpartum (Sheldon et al., 2008, 2009). On the other hand, subclinical endometritis is characterized by the presence of more than 18% of neutrophils in endometrial cytology in samples collected between 20 to 33 days postpartum or more than 10% of neutrophils at 34 to 47 days postpartum (Kasimanickam et al., 2004; Sheldon et al., 2009). Currently, the term clinical endometritis is being replaced by purulent vaginal discharge, as neutrophil infiltration in endometrium is not observed in all animals that present purulent vaginal discharge (Bogado Pascottini et al., 2023). Likewise, it is common to replace the term subclinical endometritis for cytological endometritis (Dubuc et al., 2010; McDougall et al., 2011).

In addition to clinical and subclinical hypocalcemia, there are several other risk factors for the development of clinical and subclinical endometritis, such as: parity, dystocia, twin birth, abortion, stillbirth, retained placenta, metritis, negative energy balance (NEB), ketosis, and volume of milk production (Gröhn et al., 1990; Dubuc et al., 2010). The diversity and variability of risk factors involved in the occurrence of subclinical endometritis, which are usually associated, hinder understanding the relationship among calcemia, energy metabolism and endometrial immune function. Therefore, this multivariable environment imposes a challenge to the development of more effective control and prevention strategies.

The objectives in this review are to compile information regarding the relationship between blood calcium concentrations and uterine health, as well as to discuss the available in vivo and in vitro experimental models for induced hypocalcemia and clinical or subclinical endometritis in non-transition dairy cows.

## Methodology

The research theme was established through the PICO strategy and the electronic databases selected for the research were “Google Scholar”, “Scientific Electronic Library” (Scielo) and “PubMed”. The objective was to investigate the relationship between subclinical hypocalcemia and subclinical endometritis in cattle, with emphasis on the experimental models used for induction and study of these conditions. In vivo or in vitro models with clear and reproducible experimental design were included.

To search for potentially eligible studies, the following descriptors were used: “subclinical hypocalcemia”, “experimental model”, “calcium chelation”, “EDTA”, “EGTA”, “subclinical endometritis”, “cytological endometritis”, “uterine inflammation”, “*in vitro*”, “*in vivo*”, “cattle”, “LPS” and “bovine”. After this initial screening, the references of the initially selected articles were also verified through “ResearchGate” and “Google Scholar”.

A total of 102 publications were initially identified. These studies were tabulated to assess scientific quality and risk of bias. Eligibility was confirmed by reading the articles in full, assessing the selected inclusion criteria, such as the population and the intervention performed. Study exclusion criteria included duplication of studies or data used, and methodological flaws that could compromise the reliability of the results. Thus, of the 102 studies selected in the initial screening, 57 remained.

### The role of calcium in the endometrial immune response

The mechanisms by which the immune system responds to an infection or disease are complex and can be classified as innate or adaptive. Innate immunity is nonspecific, acting in the initial response of the host upon exposure to an infectious agent. It includes physiological barriers such as the skin and mucous membranes, antimicrobial peptides, complement system and hematopoietic cells, such as monocytes, macrophages, neutrophils, eosinophils and natural killer cells (Sordillo et al., 2009; Takeuchi and Akira, 2010; Turner et al., 2012). When innate immunity is not sufficient, the adaptive immune response is activated (Janeway and Medzhitov, 2002). This response is specific for each antigenic challenge, and it can generate immunological memory (Aderem and Ulevitch, 2000; Horne et al., 2008). Adaptive immunity is classified into humoral and cellular immunity, provided by B and T lymphocytes against extracellular or intracellular pathogens, respectively (Werling and Jungi, 2003; Sordillo et al., 2009).

The pattern recognition receptors (*PRRs*) discovery raised questions regarding the classical definitions of innate and adaptive immunity, as the receptors cannot be considered nonspecific (Netea et al., 2011). Furthermore, it is known that, in situations of repeated challenges, there is an increase in the intensity of the immunological response even in mechanisms classified as innate, which was thought to be an exclusive characteristic of the adaptive response (Bowdish et al., 2007).

For an immunological response to occur, identification of the pathogen by the host is necessary (Werling and Coffey, 2007; Horne et al., 2008; Sordillo et al., 2009). After activation of *PRRs*, the production and release of chemokines, cytokines and antimicrobial peptides occur (Akira and Hemmi, 2003; Werling and Coffey, 2007; Swangchan-Uthai et al., 2012). The best-known *PRRs* are the toll-like receptors (*TLRs*) and the nucleotide binding oligomerization domain (*NOD*) or NOD-like receptors (*NLRs*) (Rietdijk et al., 2008; Takeuchi and Akira, 2010; Turner et al., 2012).

The immune response may be local or may involve the mobilization of circulating leukocytes (Sordillo et al., 2009). During infection, bacterial lipopolysaccharide (LPS) from the outer membrane of gram-negative bacteria plays an important role in recognizing and triggering the inflammatory response (Mogensen, 2009). In mammals, recognition of LPS occurs through toll-like receptor 4 (*TLR-4*) and myeloid differentiation factor 2 complex receptors (Park et al., 2009). After LPS binding to *TLR4* and myeloid differentiation factor 2 complex, factors such as nuclear factor-κB and components of the interferon-regulatory factor family are active (O'Neill and Bowie, 2007; Lu et al., 2008), resulting in production of cytokines that stimulate innate and adaptive immunity (Schroder et al., 2004). The increase in cytokines and chemokines stimulates the expression of adhesion molecules such as integrin *LFA-1* (*CD11aCD18*) and *MAC-1* (*CD11bCD18*) by neutrophils, as well as the migration of leukocytes to the site of inflammation (Harada et al., 1994). Neutrophil activation and migration are Ca-dependent processes (Clemens and Lowell, 2015); therefore, calcium levels must be within the physiological range for an adequate immune response. Furthermore, it has been demonstrated that hypocalcemic cows have increased cortisol serum levels (Horst and Jorgensen, 1982), reduced neutrophilic phagocytic activity (Martinez et al., 2012), and impaired peripheral blood mononuclear cells response to activation stimulus (Kimura et al., 2006).

A prompt and coordinated innate immune response is essential to protect the uterus from postpartum bacterial invasion. This defense relies heavily on neutrophils and macrophages, which are rapidly recruited to the endometrium through the action of proinflammatory mediators such as IL-1, IL-6, and TNF. These mechanisms work in concert to eliminate pathogens and restore uterine health; however, if impaired, they increase the risk of persistent inflammation and metritis (Bromfield et al., 2014). Calcium plays a crucial role in this process, as it not only supports uterine muscle contractility and tissue perfusion but also modulates the activation and signaling pathways of immune cells. Lower serum calcium concentrations, particularly by day 3 postpartum, have been strongly associated with increased risk of acute puerperal metritis, with higher calcium levels reducing disease incidence by up to 88% in primiparous cows (Venjakob et al., 2019).

Based on the above, there is a well established relationship between calcemia and immune response. Considering that uterine defenses are naturally weakened around parturition, subclinical hypocalcemia has been consistently linked to higher risk of uterine infections. Serum calcium concentrations below 8.59 mg/dL on at least one day within the first three days in milk (DIM) are associated with impaired neutrophil function, including reduced phagocytic activity and oxidative burst, increasing the incidence of metritis, endometritis, and fever (Martinez et al., 2012). In cows with experimentally induced subclinical hypocalcemia, lower ionized calcium concentrations in the neutrophil cytosol led to diminished phagocytic and oxidative burst responses (Martinez et al., 2014). These immunological impairments have also been linked to other periparturient disorders such as retained placenta and uterine prolapse (Hammon et al., 2006; Hansen et al., 2003; Gröhn et al., 1990; Melendez et al., 2004).

It has also been shown that hypocalcemia can vary in onset and duration depending on the cow’s lactation stage. McArt and Neves (2020) classified subclinical hypocalcemia (SCH) into three patterns—transient, persistent, and delayed—each with different health and productivity consequences. For example, cows that developed metritis tended to have average serum calcium concentrations remaining below 8.6 mg/dL until after four DIM, indicating a prolonged state of calcium deficiency associated with disease onset. Notably, cows with persistent (pSCH) and delayed (dSCH) hypocalcemia showed higher risks of disease, early culling, and decreased milk yield.

At the mechanistic level, hypocalcemia disrupts not only leukocyte counts but also the expression of genes involved in immune signaling. Hypocalcemic cows exhibit downregulation of immune genes such as *CXCL12* and *MPO*, along with reduced expression of calcium binding pathway components like *CRTAC1* and *CAMK4*, suggesting impaired intracellular signaling and reduced immune activation in polymorphonuclear leukocytes (Zhou et al., 2022). Simultaneously, upregulation of genes linked to apoptosis and cellular senescence (*CDKN1A*, *PRF1*, *HIPK3*) further compromises immune cell viability. Furthermore, lipopolysaccharide (LPS)-induced oxidative stress and apoptosis in endometrial epithelial cells have been demonstrated in vitro, underscoring how pathogen exposure can directly damage the uterine lining (Zhao et al., 2024). Collectively, these molecular, cellular, and physiological alterations contribute to an immunosuppressed state that increases susceptibility to metritis and other postpartum uterine diseases.

Although many studies have confirmed associations between hypocalcemia and various postpartum disorders, the transition period in dairy cows is highly dynamic and multifactorial, making it challenging to isolate cause-and-effect relationships. For this reason, studies using alternative models and more refined classifications of calcium status are essential to better understand the interplay between mineral metabolism, immune function, and uterine health.

### Experimental models for clinical and subclinical hypocalcemia

The peripartum period is marked by a series of homeorhetic adaptations related to nutrient mobilization and partitioning, due to increased nutritional demands from final fetal growth, colostrogenesis and early lactogenesis (Bauman and Currie, 1980). It is estimated that there is an increase in energy demand of around 250% to support lactation (Reynolds et al., 2003), while for mineral requirements, especially Ca, there is an elevation of 65% (Degaris and Lean, 2008). This substantial rise in nutritional needs is not matched by a proportional increase in ingestion capacity. As a result, most dairy cows experience a state of NEB during the initial postpartum period, leading to the mobilization of energy reserves from adipose tissue. Consequently, there is an increase in plasma levels of nonesterified fatty acids (NEFA) and β-hydroxybutyrate (BHBA; Bell, 1995). Ca excretion by the mammary gland results in a reduction in calcemia, which activates Ca homeostatic mechanisms. In response, the parathyroid glands increase the production and release of parathormone (PTH), enhancing Ca mobilization in bone, and Ca reabsorption in the kidneys. Additionally, PTH stimulates the renal production of 1,25-dihydroxy-cholecalciferol (DHCC), leading to increased intestinal Ca absorption (Goff, 2018), ultimately working to restore normocalcemia.

When the above-mentioned mechanisms to restore normocalcemia are insufficient, the animal may develop clinical or subclinical hypocalcemia. During dairy cow postpartum period, there is an increased incidence of metabolic diseases such as hypocalcemia and ketosis, besides an increased incidence of infectious diseases such as mastitis, metritis and endometritis (Neves et al., 2018), among several other metabolic and hormonal changes (Hansen et al., 2003; Ribeiro et al., 2013). This intricate relationship between energy metabolism, calcemia, and infectious diseases challenges the conclusions on results observed when the experimental model used is a dairy cow during the transition period, highlighting the importance of experimental models that induce clinical or SCH in non-transition cows. In this case, clinical or SCH might be induced with the infusion of solutions containing disodium ethylenediaminetetraacetic acid (Na_2_EDTA; Fennwick and Daniel, 1990; Jørgensen et al., 1999) or ethylene glycol tetraacetic acid (EGTA; Martinez et al., 2014).

Several studies have demonstrated the effectiveness and safety of protocols for inducing SCH using different Ca chelators (Table 1). Different Na_2_EDTA infusion rates can be safely and effectively used, to induce a state of subclinical hypocalcemia: 10.7 g/h (sufficient to chelate 57.5 mEq of Ca per hour), 7.4 g/h (sufficient to chelate 40 mEq of Ca per hour) and 5.56 g/h (sufficient to chelate 30 mEq of Ca per hour). Parathyroid hormone (PTH) secretion begins to increase rapidly after the start of the infusion with the chelator (about 15 min after the beginning of the infusion), being inversely proportional to serum Ca levels (Ramberg et al., 1967), demonstrating the importance of PTH in maintaining calcemia (Goff, 2000). In another experiment, the hypocalcemic state was obtained with the infusion of a solution containing 5% Na_2_EDTA to reach serum iCa concentration lower than 2.4 mg/dL. In the first 30 min, the infusion rate was maintained at 13 ± 1 mL/min and, subsequently, it was adjusted according to iCa levels, to maintain calcemia within the desired range. It was demonstrated that SCH depresses feed intake and rumination activity of dairy cows (Hansen et al., 2003).

**Table 1:** Experimental models for subclinical hypocalcemia induction in cattle.

**Model**	**Substance**	**Dose**	**Route**	**Major findings**	**Reference**
In vivo	Na_2_EDTA	10.7 g/h, 7.4 g/h and 5.56 g/h	IV	Decreased blood Ca and PTH response	Ramberg et al. (1967)
In vivo	10% EGTA	1.8 mg/kg/min for 7 min or 0.9 mg/kg/min for 90 min	IV	Decreased blood Ca and PTH response	Blum et al. (1978)
In vivo	5% EDTA	13 ± 1 mL/min	IV	Impaired feed intake and rumination	Hansen et al. (2003)
In vivo	5% EGTA	500 mL/h	IV	Impaired neutrophilic phagocytic and oxidative burst response	Martinez et al. (2014)
In vivo	5% EDTA	500 mL/h	IV	Decreased blood ionized and total Ca	Silva et al. (2025)

To achieve an induced subclinical hypocalcemia, in a recent study from our group conducted by Silva et al. (2025), a 5% EDTA infusion with a 500 mL/h infusion rate for approximately 45 min was performed. This protocol was effective to reduce total blood and ionized Ca. In addition, there was a reduction in blood Mg levels, probably due to a direct effect of treatment and fasting, since the animals were kept in a headlock for a period of approximately 4 h. Regarding blood metabolites, there was a significant reduction in blood BHB and there was no difference in glucose, insulin, and non-esterified fatty acids (NEFA).

Another Ca chelator efficient in inducing hypocalcemia is EGTA, which has a greater affinity for Ca at physiological pH, and low affinity for other cations (Sanui and Pace, 1967; Martinez et al., 2014; Connelly et al., 2023). The effectiveness of a 10% EGTA infusion inducing hypocalcemia was previously demonstrated in doses of 1.8 mg/kg/min for 7 min or 0.9 mg/kg/min for 90 min, both with satisfactory results. In that study, it was possible to observe an acute PTH increase (between 4 and 8 min after the infusion started) to maintain serum Ca levels (Blum et al., 1978).

Martinez et al. (2014) provided a detailed description of the methodology for preparing the solution with the chelator EGTA. A 5% EGTA solution was prepared using 900 mL of sterile water plus 50 g of EGTA and 50 mL of 5 M NaOH with an adjusted pH of 7.4. In that experiment, the infusion was carried out for 24 h. The initial infusion rate was 500 mL/h until serum iCa levels declined to less than 4 mg/dL, and then was adjusted hourly, maintaining iCa levels between 2.8 and 3.6 mg/dL, ranging from 150 to 600 mL/h (357 ± 7.2 mL/h). All cows reached SCH (iCa <4 mg/dL) in less than 2 h of infusion and only returned to normal iCa levels 6 h after the end of the infusion. The induced SCH reduced dry matter intake and rumen motility, impaired insulin release, increased lipolysis, decreased levels of iCa in the cytosol of neutrophils and, as a result, there was impairment in the neutrophil's capacity for phagocytosis and oxidative burst. Therefore, these experimental models allow the understanding of the effects of blood Ca fluctuations alone, without the metabolic and hormonal variations of the immediate postpartum period.

### Models for endometritis induction in cattle

Approximately 50% of dairy cows may develop one or more types of reproductive tract inflammation within 5 weeks postpartum (Bogado Pascottini et al., 2023). Bacterial infections cause endometritis and infertility in cattle through mechanisms related to innate immunity (Carneiro et al., 2016; Bogado Pascottini et al., 2023). To understand these mechanisms, several in vivo or in vitro models have already been used (Schmitz et al., 2004; Borges et al., 2012; Swangchan-Uthai et al., 2012; Lüttgenau et al., 2016; Moraes et al., 2017; Magalhães et al., 2022). In vivo models are generally more expensive and present greater variability according to the individual (Herath et al., 2006a). In vitro models, on the other hand, are more practical and have less variability, facilitating the study of these mechanisms, but these models can be considered reductionist, since there is no interaction with other tissues, organs and systems, as would occur in the individual.

### In vivo models for endometritis induction in cattle

Several experimental methods have already been proposed as in vivo endometritis models for cattle (Table 2). The intrauterine infusion of pathogenic bacteria, such as *Escherichia coli* (5.19 × 10^8^ CFU) and *Trueperella pyogenes* (4.34 × 10^8^ CFU) has proven effective in inducing endometrial inflammation, even causing long-term negative effects, such as reduced pregnancy rate and increased days open (Husnain et al., 2023a). The same research group also showed differences in the response to induced endometritis between multiparous and nulliparous cows. Induced endometritis in lactating multiparous cows led to more pronounced changes in histotroph composition and conceptus development, including altered gene expression related to nutrient uptake, immune response, and maternal recognition of pregnancy, while nulliparous heifers experienced similar but less severe impacts, with conceptuses showing restricted growth and activated immune responses (Husnain et al., 2023b). In another study, 10 mL of a solution containing 5.05 × 10^7^ CFU/mL *E. coli* MS499 or 10 mL of a solution containing 3.65 × 10^7^ CFU/mL *T. pyogenes* MS249 were used, both of which were effective in inducing endometritis in cattle. It was shown that the induced endometritis impaired the oocyte development capacity up to the morula stage, indicating a decrease in oocyte quality (Dickson et al., 2020). Furthermore, the use of recombinant human interleukin-8 (rhIL-8) at a dose of 5 mg per cow has already been tested, demonstrating that the model is also effective and allows polymorphonucleate transmigration assays in controlled situations (Zerbe et al., 2003). Intrauterine infusion with 10 mL of a suspension containing *E. coli* (10^6^ CFU/mL) results in increased expression of PPAR mRNA and proteins, demonstrating a role in the endometrial immune response (Socha et al., 2018). Intrauterine inoculation of *T. pyogenes* (3 × 10^9^ CFU) or β-hemolytic *E. coli* (1.5 × 10^9^ CFU) resulted in acute uterine infections (Del Vecchio et al., 1992) and, therefore, may not be suitable for an induced subclinical endometritis model at this dosage.

**Table 2:** Experimental in vivo models for subclinical endometritis in cattle.

**Model**	**Substance**	**Dose**	**Route**	**Major findings**	**References**
In vivo	*T. pyogenes* or β-hemolytic *E. coli*	3 × 10^9^ CFU or 1.5 × 10^9^ CFU respectively	intrauterine	Effects of acute uterine inflammation on estrus cycle hormonal profile	Del Vecchio et al. (1992)
In vivo	human interleukin-8	5 mg per cow	intrauterine	Polymorphonucleate transmigration assays in controlled situations	Zerbe et al. (2003)
In vivo	LPS	3 µg/kg	intrauterine	Repeated intrauterine LPS infusions alter gene expression and induce premature luteolysis	Lüttgenau et al. (2016).
In vivo	LPS	150 µg or 300 µg	intrauterine	LPS infusion stimulated circulating PMN expression of MAC-1 molecules and improved neutrophilic phagocytosis and oxidative burst	Moraes et al. (2017)
In vivo	*E. coli*	10 mL of a suspension containing 10^6^ CFU/mL	intrauterine	Increased expression of *PPAR* mRNA and proteins	Socha et al. (2018)
In vivo	*E. coli MS499* or *T. pyogenes*	10 mL of a suspension containing 5.05 × 10^7^ CFU/mL or 10 mL of 3.65 × 10^7^ CFU/mL respectively	intrauterine	Impaired oocyte development	Dickson et al. (2020)
In vivo	LPS	12.5 µg/Kg	intrauterine	Estradiol and progesterone modulate the local endometrial immune response	Magalhães et al. (2022)
In vivo	*E. coli* or *T. pyogenes*	5.19 × 10^8^ CFU and 4.34 × 10^8^ CFU respectively	intrauterine	Long-term negative reproductive effects of Induced subclinical endometritis in cattle	Husnain et al. (2023a)
In vivo	LPS	300 μg	intrauterine	Intrauterine LPS did not alter insulin gene response or inflammation in cows with or without induced subclinical hypocalcemia	Silva et al. (2025)

One alternative to the infusion of bacteria is the use of LPS. Intravenous (IV) administration of LPS in cattle results in a decrease in blood Ca levels, indicating the Ca role in inflammatory and immune response (Waldron et al., 2003; Kvidera et al., 2017). Horst et al. (2020) demonstrated that, after 12 h of the LPS challenge, there was a 23% reduction in iCa levels, requiring the infusion of 13.7 g of Ca to maintain calcemia during this period. Although calcemia reduction induced by LPS has been widely reported in several species (Elsasser et al., 1996; Carlstedt et al., 2000; Waldron et al., 2003; Toribio et al., 2005; Kvidera et al., 2017; Horst et al., 2020; Shinozuka et al., 2018; Meurer and Höcherl, 2019), the mechanisms responsible for this reduction are not yet completely understood. Some authors believe that blood Ca fluctuations can be due to the use of Ca by the activated immune system (Lewis, 2001; Kimura et al., 2006). The recruitment of leukocytes initiates a signaling cascade that results in the release of Ca from the endoplasmic reticulum to the cytosol; the decrease in intracellular Ca reserves triggers the continuous influx of extracellular Ca through the so-called store-operated Ca^2+^ (SOCE), present in the cell membrane (Lewis, 2001; Vig and Kinet, 2009; Lewis, 2020). It is likely that these mechanisms are responsible, at least in part, for calcemia fluctuations during periods of inflammation and immune response activation (Waldron et al., 2003).

Moreover, inflammation and activation of the immune system can be experimentally obtained through the administration of lipopolysaccharides (LPS) in cattle, via intravenous (Fernandes et al., 2019), intramammary (Campos et al., 2018) and intrauterine (Moraes et al., 2017) routes. Intrauterine LPS challenge is a viable and easily repeatable method for endometrial inflammatory induction in cattle. In this case, it is important to consider the estrous cycle phase, as estradiol (E2) and progesterone (P4) modulate the local endometrial immune response (Lewis, 2003). Ovariectomized cows were challenged with 12.5 µg/Kg of LPS intrauterine, and the infiltration of polymorphonuclear cells (PMN) in the endometrium after 24 h was similar to that observed in cases of endometritis, being more intense in animals previously treated with P4, compared to animals treated with E2 (Magalhães et al., 2022). Moraes et al. (2017) studied the effects of intrauterine infusion with LPS in cows with purulent vaginal discharge on the uterine expression of genes related to the inflammatory response, as well as on the effects related to the function of PMN, after 6 and 24 h of intrauterine challenge. Intrauterine infusions were performed with 20 mL of PBS containing 150 µg or 300 µg of *E. coli* LPS. Both treatments did not affect mRNA expression of endothelial leukocyte adhesion molecule (E-selectin), intracellular adhesion molecule-1 (ICAM-1), and vascular cell adhesion molecule-1 (VCAM-1), and the cytokines tumor necrosis factor-α (TNF-α), IL-1β, IL-6, IL-8, and IL-10), and TLR-4. Also, the expression of L-selectin by PMN was not affected, but there was an increase on MAC-1 expression. An increase in neutrophil phagocytosis and oxidative burst was observed in cows challenged with LPS, especially at a dose of 300 µg of LPS, compared to the control group. The authors attributed the lack of significant results to the moment of evaluation (6 and 24 h post challenge). The moment of assessment is an important variable, as demonstrated in an in vitro model, in which an increase in endometrial mRNA expression for IL-1β, IL-8, TNF, and TLR was observed after one hour of incubation with LPS and, after 12 h, the expression of TNF-α and IL-1β was significantly reduced (Swangchan-Uthai et al., 2012). In an in vivo study, intramammary challenge with LPS promoted a peak in TNF-α expression in mammary tissue within 3 h after challenge, returning to baseline values after 12 h (Schmitz et al., 2004). In an attempt to better explore the relationship between calcemia and endometritis, our group performed an intrauterine infusion of 300 μg LPS in cows with or without subclinical hypocalcemia and after 3 h, endometrial biopsies were collected to determine the expression of *IL-6*, *CXCL8*, *TNF* and gene expression of glucose-insulin receptors and insulin-related genes (Silva et al., 2025). No significant difference was observed between the treatment and control groups, which may be explained by the short-term induced hypocalcemia. Unfortunately, only a single uterine sample collection was performed because the sample collection itself can result in uterine inflammation (Lüttgenau et al., 2016). Therefore, although induced hypocalcemia allows evaluating the relationship between calcemia and uterine inflammation, its transient duration does not mimic the duration of hypocalcemia experienced by postpartum cows. Therefore, more studies are needed to determine the ideal moment of evaluation for each cytokine, model and species studied.

### In vitro models for endometritis induction in cattle

The uterine endometrium is composed of epithelial, stromal, endothelial cells and cells of the immune system, which are modulated by hormones such as estradiol and progesterone (Lewis, 2003), in addition to growth factors, cytokines and chemokines. These autocrine, paracrine and endocrine modulations are fundamental to the normal physiology of the tissue and cannot be reproduced in its entirety using in vitro models (Borges et al., 2012). In the case of in vitro epithelial and stromal cell culture, endometrial tissue is disrupted (mechanically or enzymatically) as a way of increasing the perfusion of oxygen and nutrients in the culture medium, resulting in the loss of the tissue's normal architecture (Fortier et al., 1988). In this process, there is also the release of damage-associated molecular patterns (DAMPs), which will probably result in sterile inflammation, compromising data related to endometrial inflammation (Chen and Nuñez, 2010; Cukierman et al., 2001; Vogel and Sheetz, 2006). An alternative is the use of ex vivo models of bovine endometrium with explants obtained through endometrial biopsy. In this case, the explants have lower basal levels of interleukins compared to methods that use chopped tissue. Although promising, it is still possible to observe significant variations between endometrial explants according to the estrous cycle phase when it was obtained, thus this must be considered when interpreting in vitro data (Borges et al., 2012).

Several in vitro and *ex vivo studies* have been developed to investigate the mechanisms underlying endometrial inflammation in cattle (Table 3). One of the most used strategies is the stimulation of endometrial epithelial or stromal cells with bacterial components, particularly LPS. Both epithelial and stromal cells from bovine endometrium express Toll-like receptor 4 (TLR4) and respond to LPS challenge with increased expression of pro-inflammatory cytokines such as *IL-1β*, *IL-6*, and *IL-8* (Herath et al., 2006b). Similarly, Swangchan-Uthai et al. (2012) showed that LPS challenge increased mRNA expression of *TNF-α* and *IL-1β* in bovine endometrial explants within 1 h, followed by downregulation at 12 h, highlighting the time-dependent dynamics of endometrial immune response.

**Table 3:** Experimental in vitro models for subclinical endometritis in cattle.

**Model**	**Substance**	**Dose**	**Major findings**	**References**
In vitro *and ex vivo*	LPS and *E. coli*	0.03–1 g/mL LPS or 10^2^–10^5^ CFU/mL *E. coli* for 24 h	Bacteria modulate epithelial and stromal cell’s endocrine function	Herath et al. (2006b)
In vitro	LPS	1 μg/mL for 24 h	Epithelial cells have an essential role in the innate immune defense of the bovine endometrium	Davies et al. (2008)
In vitro *and ex vivo*	LPS	1 or 3 µg/mL for 24 h	LPS detection by endometrial cells stimulated the accumulation of PGE rather than PGF_2α_	Herath et al. (2009)
Ex vivo	*E. coli or T. pyogenes*	1 × 10^8^ CFU/mL for 24 and 48 h	ex vivo model of intact endometrium to explore the mechanisms of immunity and inflammation	Borges et al. (2012)
In vitro	LPS	1 µg/mL for 6 h	LPS recognition occurs via TLR4- and MYD88 signaling pathways	Cronin et al. (2012)
In vitro	LPS or *E. coli*	1 mg/mL LPS or 10^3^ CFU/mL *E. coli* for 24 h	The stage of the estrus cycle or ovarian steroids did not modulate the innate immune response	Saut et al. (2014)
In vitro	LPS	100 ng/mL for 0, 1, 6, 12, 24, or 48 h	LPS upregulated known target genes through the TLR4/CD14/MD2 complex signaling pathway	Swangchan-Uthai et al. (2012)
In vitro	LPS	100 ng/mL for 24 h	Link between TLR2, TLR1, and TLR6 responses and bacterial infections	Turner et al. (2014)
In vitro	LPS	100 ng/mL for 6 h	A response to infection may interfere with the establishment of pregnancy	Oguejiofor et al. (2015)
In vitro	*E. coli*	10^6^ CFU/mL for 2, 12 and 24 h	Leukotrienes enhance experimentally induced infection	Korzekwa et al. (2016)
In vitro	LPS	10 or 100 ng/mL for 1, 6, 12, 24 and 48 h	Potential treatment of endometritis with platelet-rich plasma	Marini et al. (2016)
In vitro	LPS	0, 8, and 16 µg/mL LPS for 72 h	Changes in protein expression profiles in bovine Endometrial Epithelial Cells	Piras et al. (2017)
In vivo	LPS	0, 2, 4, 8, 12, 16 or 24 g/mL for 72 h	Model for mechanisms by which LPS deregulates endometrial function	Chanrot et al. (2017)
In vitro	LPS	100 ng/mL for 6 h	RsLPS may act as antagonist for TLR4 in bovine endometrial cells	Chotimanukul et al. (2017)
In vitro	LPS	1, 5, 10, and 20 µg for 48 h	CCL2 may support maternal-fetal interface	Lim et al. (2018)
In vitro	LPS	100 ng/mL for 24 h	Negative energy balance may be linked to postpartum uterine disease	Noleto et al. (2017)
In vitro	LPS	1 μg/mL for 0, 3, 12, or 18 h	Cortisol may exert its anti-inflammatory actions by regulating NF-κB activation and MAPK phosphorylation	Dong et al. (2018)
In vitro	LPS	1 μg/mL for 6h	bEEL and bCSC cell lines are excellent in vitro models for intrauterine environment studies	Almughlliq et al. (2018)
In vitro	LPS	1 μg/mL	IL-1β may act unusually in an autocrine-positive feedback loop	Koh et al. (2018)
In vitro	LPS	100 ng/mL for 4, 8 and 12 h	LPS-induced inflammatory responses could be downregulated by exogenous PGE_2_ but not PGF_2α_	Shen et al. (2018)
In vitro	LPS	1 µg/mL for 12, 24, 48, 72 and 96 h	Participation of *PPAR* receptors in signal transduction during LPS induced inflammation in endometrium	Socha et al. (2018)
In vitro	LPS	1 μg/mL LPS for 24 h	LPS induces the expression of *IL-6* and *IL-8* mRNA	Wang et al. (2018)
In vitro	LPS	100 ng/mL for 0, 2, 4, 8, 16, and 24 h	PGE_2_ Balance may improve recovery from endometrial injuries	Deng et al. (2019)
In vitro	LPS	0, 2, and 8 μg/mL for 24 h	Possible long-term effects of inflammation on endometrial function and fertility	Guo et al. (2019)
In vitro	LPS	1 μg/mL for 12 h	Ferulic acid may be considered as a potential anti-inflammatory drug for endometritis	Yin et al. (2019)
In vitro	*E. coli* or LPS	1 × 10^6^ CFU/mL live *E. coli*, 10^8^ CFU/mL heat-killed *E. coli*, or 1 μg/mL LPS	Cortisol exerted anti-inflammatory effects	Cui et al. (2020b)
In vitro	LPS	0.5 µg/mL for 12 h	Possible molecular mechanisms of microbial invasion and host cell response	Ding et al. (2020)
In vitro	LPS	LPS 1 µg/mL for 6 h	Protective effect of Hydroxytyrosol in the treatment and prevention of uterine pathologies in cows	Gugliandolo et al. (2020)
In vitro	LPS	2 or 8 μg/mL LPS for 24 h	LPS activates pro-inflammatory mechanisms leading to perturbed immune balance and cell adhesion processes	Jhamat et al. (2020)
In vitro	*E. coli* or LPS	*E. coli* (2 × 10^6^ or 2 × 10^7^ CFU/mL) for 2 h or LPS at 0, 3, or 10 mg/mL for 24 h	Decreased S100A4 expression during endometrial inflammation	Li et al. (2020)
In vitro	*E. coli* or LPS	1 × 10^5^ CFU/mL *E. coli* or 1 μg/mL LPS for 6 or 24 h	Progesterone has anti-inflammatory effect, inhibiting NF-κB activation and MAPK phosphorylation	Cui et al. (2020a)
In vitro	LPS	1 μg/mL for 6 h	Astaxanthin counteracts LPS-induced inflammation, oxidative stress, apoptosis, and tight junction disassembly	Wan et al. (2020)
In vitro	LPS	1 μg/mL LPS for 4, 12, and 24 h	β-endorphins may inhibit the endometrial inflammatory response through δ opioid receptor	Cui et al. (2021)
In vitro	LPS	100 ng/mL for 15, 30, or 60 min	PGE_2_ accumulation strengthened inflammation	Deng et al. (2021)
In vitro	LPS	0.1, 1, and 10 µg/mL for 6 h	Inhibitory role of bovine adipose-derived mesenchymal stem cells in inflammation	Lu et al. (2021)
In vitro	LPS	0, 3, 10, and 30 µg/mL for 24 h	LPS could cause inflammation and mediate pyroptosis through classical and nonclassical inflammasome pathways	Ma et al. (2021)
In vitro	LPS	1 µg/mL for 8 h	Estrous cycle phase altered endometrial gene expression profiles	Ault-Seay et al. (2022)
In vitro	*LPS or E. coli*	1 μg/mL or 10^8^ CFU/mL, respectively	Anti-inflammatory effect of progesterone probably mediated through MAPK and NF-κB pathways	Cui et al. (2022)
In vitro	LPS	0, 2, and 8 µg/mL for 24 h	Mechanistic explanation for the relationship between LPS and cell proliferation	Najafi et al. (2022)
Ex vivo	LPS	1 μg/mL of LPS for 24 h	Eicosapentaenoic acid decreases pro-inflammatory cytokines	Carneiro et al. (2023)
In vitro	LPS	1 µg/mL for 4 h	A20 expression was upregulated in the bovine endometrial epithelial cells following LPS stimulation	Dong et al. (2024)
In vitro	LPS	1 μg/mL for 24 h	Berberine reduces ROS levels by upregulating the Nrf2 pathway.	Guo et al. (2024)
In vitro	LPS	5 µg/mL LPS for 6 h	5 µg/mL LPS for 6 h could be used as an inflammation model	Li et al. (2024a)
In vitro	LPS	10 µg/mL LPS for 24 h	Se can attenuate LPS-induced damage and promote cell proliferation and migration in vitro	Li et al. (2024b)
In vitro	LPS	10 µg/mL for 24 h	Cecropin A can effectively alleviate LPS-induced cell apoptosis	Zhao et al. (2024)

Further studies have used endometrial explants or cell lines to evaluate inflammatory modulation under different hormonal or pharmacological conditions. Borges et al. (2012) used bovine endometrial explants to investigate innate immune responses and demonstrated that explants preserve the structural integrity of the tissue better than dissociated cultures, allowing more physiological insights into cytokine production. Marini et al. (2016) developed an ex vivo inflammatory model to test the effects of platelet-rich plasma on LPS-induced endometrial inflammation and reported a reduction in *TNF-α* and *IL-8* levels, suggesting potential therapeutic applications. Other studies, explored the role of prostaglandin E2 (PGE_2_) in modulating LPS-induced damage in endometrial explants, emphasizing the link between inflammation and tissue homeostasis (Deng et al., 2019; Deng et al., 2021).

Recent investigations have also applied these models to assess the effects of pharmacological agents and endocrine modulators. Carneiro et al. (2023) evaluated the influence of omega-3 fatty acids on bovine endometrial explants challenged with LPS and observed changes in *IL-10* and *IL-1β* expression, indicating immunomodulatory potential. Likewise, Cui et al. (2021) tested the effect of β-endorphin via δ-opioid receptor signaling and demonstrated a reduction in the expression of inflammatory mediators in LPS-stimulated endometrial cells. These findings illustrate how in vitro models are valuable tools for dissecting cellular and molecular responses to inflammatory stimuli and may support the development of new strategies for the control of uterine diseases in dairy cattle.

## Conclusions

Subclinical hypocalcemia is a very prevalent disease during the postpartum period and is an important predisposing factor to the occurrence of endometritis in dairy cows. However, the study of the pathophysiology of diseases in the postpartum period of dairy cows is very challenging, due to the complexity and large number of alterations that occur during this period. Experimental models that artificially induce subclinical hypocalcemia or endometrial inflammation, especially in non-transition cows, allow for better control of confounding variables. Current models still face limitations and, therefore, future efforts to the development and refinement of in vivo and in vitro experimental models are necessary.

## Data Availability

No research data was used.
